# GHANAIAN NURSING STUDENTS’ EXPERIENCES IN CARING FOR OLDER ADULTS IN ACUTE CARE SETTINGS

**DOI:** 10.1093/geroni/igae098.1876

**Published:** 2024-12-31

**Authors:** Diana Abudu-Birresborn, Martine Puts, Lynn McCleary, Charlene Chu, Vida Yakong, Lisa Cranley

**Affiliations:** Brock University, St. Catharines, Ontario, Canada; University of Toronto, Toronto, Ontario, Canada; Brock University, St. Catharines, Ontario, Canada; University of Toronto, Toronto, Ontario, Canada; University for Development Studies, Tamale, Northern, Ghana; University of Toronto, Toronto, Ontario, Canada

## Abstract

Evidence suggests that nursing students’ experiences in acute care settings may negatively impact their views on caring for older adults, resulting in poor interest and quality of care. However, Ghanaian nursing students’ experiences caring for older adults are unknown. We aimed to explore students’ experiences in caring for older adults to help develop and improve strategies, that promote students’ positive experiences and promote quality of care for older adults. We employed a descriptive qualitative approach and interviewed 17 second and third-year nursing students since they had clinical exposure. Interviews were conducted face-to-face and via telephone from November 2020- December 2020 and lasted 45-60 minutes. Interviews were audio recorded, transcribed, coded, and sorted with Nvivo. Data were analyzed and interpreted, themes were generated using critical thematic analysis and contextual meanings students ascribed to their responses. Two broad themes were generated. 1) experiences in caring for older adults and 2) inadequate staff and resources. Students had challenging experiences caring for older adults who were dependent and unable to manage their activities of daily living. They described caring for older adults as physically and emotionally demanding. They noted that limited staff and resources constrained caring for older adults which frustrated them when providing care. However, they expressed caring for older adults as a positive learning experience as they perceive them as a source of wisdom and blessings. Nurse leaders and administrators can help shape students’ experiences caring for older adults by ensuring adequate staff and resources and supporting students’ clinical learning.

